# Supplementary material for: Spatial inequities in access to medications for treatment of opioid use disorder highlight scarcity of methadone providers under counterfactual scenarios
Source: PLoS Comput Biol. 2024 Jul 26;20(7):e1012307. doi: 10.1371/journal.pcbi.1012307 (PMC11305545; doi:10.1371/journal.pcbi.1012307)
Supplement: S1 Supporting Information — Table A. Overall syringe sharing reduction and regret scores under for each reasonable access assumption under each scenario. Table B. Overall syringe sharing reduction relative risk and regret scores for each reasonable access assumption under each scenario. (DOCX) [file pcbi.1012307.s001.docx]

**Spatial inequities in access to medications for treatment of opioid use disorder highlight scarcity of methadone providers under counterfactual scenarios – S1 Supporting Information**

**Table A. Overall syringe sharing reduction and regret scores under for each reasonable access assumption under each scenario.**

| **Overall Needle Sharing Reduction (Enrollment 90% comapred to Enrollment 0%) & Regret Scores** | | | | | | | | | | | |
| --- | --- | --- | --- | --- | --- | --- | --- | --- | --- | --- | --- |
| Threshold | Penalty | Max dist threshold | **Needle sharing reduction** | | | | **Best (max)** | **Regret score** | | | |
|  |  |  | Actual | Spatially Random | Need-based 1 | Need-based 2 |  | Actual | Spatially Random | Need-based 1 | Need-based 2 |
| 1/5 | 0.6 | F | 870,055 | 428,789 | 943,217 | 1,013,127 | 1,013,127 | 143,072 | 584,338 | 69,910 | 0 |
| 1/5 | 0.6 | T | 869,749 | 428,789 | 943,217 | 1,015,941 | 1,015,941 | 146,191 | 587,152 | 72,723 | 0 |
| 1/5 | 0.75 | F | 894,871 | 485,978 | 972,420 | 1,043,158 | 1,043,158 | 148,287 | 557,181 | 70,738 | 0 |
| 1/5 | 0.75 | T | 902,369 | 485,978 | 972,420 | 1,034,218 | 1,034,218 | 131,849 | 548,241 | 61,798 | 0 |
| 1/5 | 0.9 | F | 985,347 | 655,979 | 1,036,241 | 1,097,266 | 1,097,266 | 111,919 | 441,287 | 61,026 | 0 |
| 1/5 | 0.9 | T | 980,194 | 655,979 | 1,036,241 | 1,086,808 | 1,086,808 | 106,613 | 430,829 | 50,567 | 0 |
| 2/10 | 0.6 | F | 1,014,184 | 713,252 | 1,107,983 | 1,049,910 | 1,107,983 | 93,799 | 394,731 | 0 | 58,073 |
| 2/10 | 0.6 | T | 1,006,731 | 713,252 | 1,107,983 | 1,061,798 | 1,107,983 | 101,252 | 394,731 | 0 | 46,185 |
| 2/10 | 0.75 | F | 1,016,346 | 734,274 | 1,119,774 | 1,062,995 | 1,119,774 | 103,427 | 385,500 | 0 | 56,779 |
| 2/10 | 0.75 | T | 1,019,656 | 734,274 | 1,119,774 | 1,063,299 | 1,119,774 | 100,118 | 385,500 | 0 | 56,474 |
| 2/10 | 0.9 | F | 1,050,250 | 822,836 | 1,116,509 | 1,077,796 | 1,116,509 | 66,259 | 293,673 | 0 | 38,713 |
| 2/10 | 0.9 | T | 1,044,803 | 822,836 | 1,116,509 | 1,075,730 | 1,116,509 | 71,706 | 293,673 | 0 | 40,779 |
| 5/20 | 0.6 | F | 1,058,847 | 807,050 | 1,072,603 | 1,042,241 | 1,072,603 | 13,757 | 265,553 | 0 | 30,362 |
| 5/20 | 0.6 | T | 1,061,379 | 807,050 | 1,072,603 | 1,044,011 | 1,072,603 | 11,225 | 265,553 | 0 | 28,592 |
| 5/20 | 0.75 | F | 1,061,969 | 825,601 | 1,079,210 | 1,051,867 | 1,079,210 | 17,242 | 253,609 | 0 | 27,343 |
| 5/20 | 0.75 | T | 1,071,019 | 825,601 | 1,079,210 | 1,046,988 | 1,079,210 | 8,192 | 253,609 | 0 | 32,222 |
| 5/20 | 0.9 | F | 1,064,993 | 896,101 | 1,082,641 | 1,059,959 | 1,082,641 | 17,648 | 186,540 | 0 | 22,682 |
| 5/20 | 0.9 | T | 1,072,466 | 896,101 | 1,082,641 | 1,053,021 | 1,082,641 | 10,175 | 186,540 | 0 | 29,620 |
|  |  |  |  |  |  |  | 75th percentile | 110,593 | 438,673 | 58,411 | 40,262 |

Table A shows the syringe sharing reduction outcome for each reasonable access assumption under each scenario, along with regret scores. For example, when the ideal geographical travel distance preference is set to be low (i.e., 1 mile for urban and 5 miles for suburban), penalty equals to 0.6, and we do not set a maximum limit, Need-based 2 generates the most syringe sharing reduction (1,013K, see first row in Table A). Accordingly, the regret score for each other scenario is the difference between their syringe sharing reduction result and Need-based 2. In this case, spatially random generates the largest regret score, meaning we expect the syringe sharing reduction to be the lowest in this case. In the last row of Table A, we report the 75th percentile of regret scores across each of the 18 reasonable access assumptions for each scenario, from which we observe that the two *Need-based* scenarios perform the best. Notably, the *Actual* scenario performs worse than the two *Need-based* scenarios but better than the *Random* scenario.

**Table B. Overall syringe sharing reduction relative risk and regret scores for each reasonable access assumption under each scenario.**

| **Overall Needle Sharing Reduction Relative Risk (Enrollment 90%/Enrollment 0%, zip code weighted by Enrollment 0%)** | | | | | | | | | | | | |
| --- | --- | --- | --- | --- | --- | --- | --- | --- | --- | --- | --- | --- |
| Experiment |  | Penalty | Threshold | Actual | Spatially Random | Need-based 1 | Need-based 2 | Best (min) | Actual | Spatially Random | Need-based 1 | Need-based 2 |
| 1 | No Max  Distance Threshold | 0.6 | 1/5 | 0.567 | 0.786 | 0.530 | 0.495 | 0.495 | 0.071 | 0.291 | 0.035 | 0.000 |
| 2 |  | 0.6 | 2/10 | 0.495 | 0.645 | 0.448 | 0.477 | 0.448 | 0.047 | 0.197 | 0.000 | 0.029 |
| 3 |  | 0.6 | 5/20 | 0.472 | 0.598 | 0.466 | 0.481 | 0.466 | 0.007 | 0.132 | 0.000 | 0.015 |
| 4 |  | 0.75 | 1/5 | 0.554 | 0.758 | 0.516 | 0.480 | 0.480 | 0.074 | 0.278 | 0.035 | 0.000 |
| 5 |  | 0.75 | 2/10 | 0.494 | 0.634 | 0.442 | 0.470 | 0.442 | 0.052 | 0.192 | 0.000 | 0.028 |
| 6 |  | 0.75 | 5/20 | 0.471 | 0.589 | 0.462 | 0.476 | 0.462 | 0.009 | 0.126 | 0.000 | 0.014 |
| 7 |  | 0.9 | 1/5 | 0.509 | 0.673 | 0.484 | 0.453 | 0.453 | 0.056 | 0.220 | 0.030 | 0.000 |
| 8 |  | 0.9 | 2/10 | 0.477 | 0.590 | 0.444 | 0.463 | 0.444 | 0.033 | 0.146 | 0.000 | 0.019 |
| 9 |  | 0.9 | 5/20 | 0.469 | 0.554 | 0.461 | 0.472 | 0.461 | 0.009 | 0.093 | 0.000 | 0.011 |
| 10 | Max 15/60 miles Distance Threshold | 0.6 | 1/5 | 0.567 | 0.786 | 0.530 | 0.494 | 0.494 | 0.073 | 0.293 | 0.036 | 0.000 |
| 11 |  | 0.6 | 2/10 | 0.498 | 0.645 | 0.448 | 0.471 | 0.448 | 0.050 | 0.197 | 0.000 | 0.023 |
| 12 |  | 0.6 | 5/20 | 0.471 | 0.598 | 0.466 | 0.480 | 0.466 | 0.006 | 0.132 | 0.000 | 0.014 |
| 13 |  | 0.75 | 1/5 | 0.550 | 0.758 | 0.516 | 0.485 | 0.485 | 0.066 | 0.273 | 0.031 | 0.000 |
| 14 |  | 0.75 | 2/10 | 0.492 | 0.634 | 0.442 | 0.470 | 0.442 | 0.050 | 0.192 | 0.000 | 0.028 |
| 15 |  | 0.75 | 5/20 | 0.466 | 0.589 | 0.462 | 0.478 | 0.462 | 0.004 | 0.126 | 0.000 | 0.016 |
| 16 |  | 0.9 | 1/5 | 0.512 | 0.673 | 0.484 | 0.459 | 0.459 | 0.053 | 0.215 | 0.025 | 0.000 |
| 17 |  | 0.9 | 2/10 | 0.479 | 0.590 | 0.444 | 0.464 | 0.444 | 0.036 | 0.146 | 0.000 | 0.020 |
| 18 |  | 0.9 | 5/20 | 0.466 | 0.554 | 0.461 | 0.475 | 0.461 | 0.005 | 0.093 | 0.000 | 0.015 |
|  |  |  |  |  |  |  | 75th percentile RS | | 0.055 | 0.219 | 0.029 | 0.020 |
