# Supplementary material for: Spatial inequities in access to medications for treatment of opioid use disorder highlight scarcity of methadone providers under counterfactual scenarios
Source: PLoS Comput Biol. 2024 Jul 26;20(7):e1012307. doi: 10.1371/journal.pcbi.1012307 (PMC11305545; doi:10.1371/journal.pcbi.1012307)
Supplement: Attachment — Submitted filename: MethadoneAccessReview.pdf [file pcbi.1012307.s002.pdf]

Thank you for the opportunity to review this examination of travel distance scenarios to methadone treatment and the associated influence of syringe sharing risks mediated by an ABM. I really like how the paper makes the benefits of spatial accessibility to methadone more tangible by quantifying reduction in risky behavior and showing how those benefits are located rather tightly around the resources. This is an important *so what* to take away from the research. The use of counterfactual scenarios also really helped to highlight the importance of strategic placement of MOUD resources. I think this work adds value to MOUD accessibility literature, and from a methodological standpoint, the integration of the ABM + accessibility analysis is instructive and creative.

One overarching concern I have for the narrative of this paper is the rather simplistic link that is made between spatial access/low distance and treatment benefits. While I realize the authors are likely trying to showcase possible benefits, I think it is important to address the substantial friction between people (particularly minority population groups) and treatment utilization, so the approaches presented in this paper are situated appropriately within the MOUD problem space.

For example, important minority access issues to methadone treatment are mentioned starting on line 89. However, the statement "*though once engaged in treatment*, have similar retention rates to the majority of clients (Elwy et al. 2008)" could mask or misconstrue access issues experienced by minority populations that influence and create substantial inequities of minority populations' induction, adherence, and treatment completion rates when compared to the White population. Guerrero et al. (2022) provides a good overview of this.

Guerrero, Erick G., Hortensia Amaro, Tenie Khachikian, Mona Zahir, and Jeanne C. Marsh. "A Bifurcated Opioid Treatment System and Widening Insidious Disparities." *Addictive Behaviors* 130 (July 1, 2022): 107296. https://doi.org/10.1016/j.addbeh.2022.107296.

Building on this, recent work (Mitchell et al., 2023) suggests that the availability of accessible MOUD providers is not the barrier to MOUD access for urban Black communities—given that consistently across the US at census tract level Black urban populations have substantially greater accessibility to MOUD than White urban communities but Black communities have worse outcomes as highlighted above by Guerrero. Additionally, the literature has documented how more affluent communities see these treatment resources as negative assets, driving the NIMBY phenomenon to keep these facilities out of their communities.

Mitchell, Penelope, Kevin M. Curtin, and Nicholas R. Magliocca. "Race, Rurality and Geographic Accessibility to Medication for Opioid Use Disorder in the U.S." *Journal of Maps* 19, no. 1 (December 31, 2023): 2270632. https://doi.org/10.1080/17445647.2023.2270632.

While it is not the job of this article to reconcile these complex accessibility and access problems, these issues should at least me noted and mentioned as limitations.

Other notes—

- Methods (lines 191-192)—it would be helpful if how distances were calculated (network vs Euclidean) was more clearly stated since the distance thresholds in the analysis are pretty small.
- Data discussion (starting at line 424)—I like how this study identifies that targeting the demand population of PWID, as opposed to the general adult population, better offsets syringe exchange. This provides valuable insights in future intervention development—one of the goals of the research. However, much of the PWID data came from syringe service programs which are not available everywhere. Could the authors speak to the challenges of characterizing the PWID population in areas where this type of data is not available to help highlight the value added by this data.
