# Supplementary material for: Spatial inequities in access to medications for treatment of opioid use disorder highlight scarcity of methadone providers under counterfactual scenarios
Source: PLoS Comput Biol. 2024 Jul 26;20(7):e1012307. doi: 10.1371/journal.pcbi.1012307 (PMC11305545; doi:10.1371/journal.pcbi.1012307)
Supplement: Attachment — Submitted filename: PLOS Comp Biol Cover letter response.pdf [file pcbi.1012307.s003.pdf]

Eric Tatara, PhD, PE

Senior Scientist at Large
Consortium for Advanced Science and Engineering
University of Chicago
Chicago, IL

Software Engineer
Decision and Infrastructure Sciences Division
Argonne National Laboratory
9700 South Cass Avenue
Lemont, IL 60439

1-312-375-0419 phone
tatara@anl.gov

April 29, 2024

Editors, PLOS Computational Biology

On behalf of my co-authors, I am pleased to submit our revised manuscript "Spatial inequities in access to medications for treatment of opioid use disorder highlight scarcity of methadone providers under counterfactual scenarios" for consideration in *PLOS Computational Biology* as a research article.

We gratefully thank and acknowledge the reviewers and Editor for their helpful comments and suggestions. We have included and addressed all the reviewer comments one-by-one below and in the attached revised manuscript.

Thank you for considering our manuscript. We are happy to further answer questions and comments from the editors and reviewers.

Sincerely,

Eric Tatara

Authors Response below in red text.

From the Editors:

The Authors are expected to address all the criticisms by all Reviewers. In particular the authors:

- clarify and assess the use of Euclidean distance on the findings (Reviewers #1 & #2),
    - Please see below direct response to Reviewers 1 & 2.
- clarify the source of zip code and spatial data (Reviewer #1),
    - Please see below direct response to Reviewers 1.
- address the substantial friction between people (particularly minority population groups) and treatment utilization
    - Please see below direct response to Reviewers 2.
- discuss the challenge on PWID data availability (Reviewer #2).
    - Please see below direct response to Reviewers 2.

In additional to the above comments, please address:

1. Figure 3, could the authors provide an explanation why the regret score showed a very different relation between travel distance preference and regret score for Need-based 2 compare to the other three scenario? (e.g. highest regret score 2mi urban / 10mi non-urban)
    - We have included a paragraph in the Results after Figure 3 to help address this.
2. Also, could the authors comment on whether mid-distance preference (2mi urban / 10mi non-urban) having the highest regret score is a general result for Need-based 2, or is a finding due to the distribution of the population/PWID in Chicago?
    - We have included a paragraph in the Results after Figure 3 to help address this.
3. Could the authors comment on the generalizability of the study findings, such as its applicability in other cities in the US, or in less urbanized areas?
    - Thanks for this question! We have added the following to Discussion:

      "While each context is uniquely different, some of the core metrics that impact treatment availability, namely density of providers and travel to clinic, can be applied in rural, urban and other diverse contexts. Future work should expand the model to other locales to ensure that assumptions around factors that drive access to care are consistent, and how any heterogeneity might be explained."
4. Following on Reviewer #2's comment, if PWID data is limited or unavailable, what would be the best strategy?
    - Please see below direct response to Reviewers 2.

Reviewer #1

The manuscript focuses on modeling MOUD access among PWID individuals. This is a very important topic, and I applaud the authors for focusing their research efforts on this area. The agent-based modeling approach looks solid, but there are a few questions that need to be addressed:

- It is not clear where the zip code and spatial data came from. For example, in 2010, per the US Census, Illinois had 1,384 zip code tabulation areas vs the 1085 "zip codes" noted in the paper. https://www.census.gov/geographies/reference-files/2010/geo/state-local-geo-guides-2010/illinois.html

    - We have included additional clarifying language in the Section on Spatial Distribution of Methadone Providers, along with the source of data (U.S. Census). The original text may have been confusing. The model study geographic area includes Cook Co, IL and surrounding collar counties, which contain 298 zip codes, which we used from 2010 Census Data (https://www.census.gov/geographies/mapping-files/time-series/geo/tiger-line-file.html) In the section Spatial Distribution of Methadone Providers, we reference the 1,085 zip codes for other areas of IL, not including the 298 for Cook and collar counties. The sum of all zip codes is 1,084.

    - We have included additional data sources in Methods:
        - Spatial boundary and clinical data used in the analysis can be found in the Opioid Environment Policy Scan Data Warehouse, version 1.0;
        - Susan Paykin, Dylan Halpern, Qinyun Lin, Moksha Menghaney, Angela Li, Rachel Vigil, Margot Bolanos Gamez, Alexa Jin, Ally Muszynski, and Marynia Kolak. (2022). GeoDaCenter/opioid-policy-scan*: Opioid Environment Policy Scan (OEPS) Data Warehouse (v1.0).* Zenodo.https://doi.org/10.5281/zenodo.5842465

- The paper notes that "Most existing studies focus on actual distance to MOUD locations, and very few have studied what is the ideal distance (or travel time) preferences to ensure accessibility.". While preference is indeed important to model, so is accurate modeling of the actual distances. This is the biggest shortcoming I noticed within this analysis. The R package sf results in Euclidean distance between two lat/long locations and not a routable distance. Many of the modeled locations end up being in Chicago, which is split by multiple rivers and interstates. Novaes and Valente, Love et al, and others have shown that routable distances vs Euclidean distances can vary from 1.2 to 1.4 (typically), depending on rural/urban and other factors. An "adjustment" was stated to have been made in urban areas from 1 mile to 2 miles, but this adjustment was made solely on the scarcity of methadone clinics.

    - This is an important contribution and we have added this to the Discussion and Limitations, specifically we included the following text:

“Travel distance and time to providers was calculated using Euclidean distance between PWID’s residence zip centroid and the nearest methadone provider location. The HepCEP model does not model travel network routing of PWID between residence and methadone provider via roads or sidewalks, which would account for longer travel distance. An explicit model of travel network routing would need to account for available modes of transportation (car, rail, bus, walking) to each PWID and is beyond the scope of this modeling effort. However, the use of the travel distance penalties described in the Reasonable Geographic Access Assumptions Section does account for uncertainties around individuals’ probability of obtaining treatment based on travel distance thresholds.”

Further, we have adjusted the text in the Reasonable Geographic Access Assumptions Section to read:

“To approximate reasonable geographic access, the travel distance in miles from zip code centroid to the nearest methadone provider is calculated using the sf package in R (version 4.0.2) [39] which provides a Euclidean distance metric. Travel network routing is not modeled in this analysis.”

- Table 1 on page 8 does not seem to show the 2-mile adjustment in urban areas that is then noted on page 9.

    - This was somewhat confusion as written. We have added clarifying text after Table 1 in the Section Reasonable Geographic Access Assumptions.

- It is not clear whether the modeling accounts for persons who start treatment but drop out. The paper notes “every 7 days” and the average treatment duration to be 150 days. However, did the model implement a normal bell-shaped distribution of treatment duration or peg all the persons modeled to be at 150 days?

    - We have added the following text to the Section Reasonable Geographic Access Assumptions:

      “Average treatment duration for methadone is obtained from literature to be 150 days [38] and the probability to start and continue treatment every 7 days is estimated using a random empirical distribution with mean of 150 days, resulting in some PWID having longer or shorter time on treatment. PWID who discontinue methadone treatment may reinitiate treatment later during the course of the simulation.”

- MOUD laws vary significantly from state to state. Illinois is one of only 8 states that require a government ID to take part in an opioid treatment program. Not all PWID

persons may fit that or other requirements. The paper should, at a minimum, speak to the wide variety of laws that differ from state to state. From a broader applicability perspective, this is important. https://www.pewtrusts.org/en/research-and-analysis/issue-briefs/2022/09/overview-of-opioid-treatment-program-regulations-by-state

- This is an important contribution and we have added this to the Discussion and Limitations, specifically we included the following text:

  "The model has several limitations around practical access to methadone treatment. First, laws around access to methadone and OTP services can vary greatly by state, with some states like Illinois requiring a government issued ID for access to services [50]. The HepCEP model does not account for inequity in access to OTPs based on an individual's possession of a valid government issued ID card. Minoritized groups, especially those who are unhoused, recently released from carceral settings, or undocumented, face additional barriers to obtaining methadone treatment for OUD due to lack of government issued ID documents. Second, the model assumes that there is no treatment capacity constraint on individual providers, e.g. every PWID who seeks methadone treatment is able to do so. OTPs across the US have experienced constraints in providing care due in part to governmental regulations and lack of coverage via private health insurance for methadone treatment [51]. In light of this, our results may overestimate access to methadone treatment based on the study locale."

- Additional clarifications are needed from a modeling MOUD distribution perspective about any assumptions that were made as to a location's ability to service a maximum number of individuals. Facilities of all types have their maximum service abilities, and MOUD locations can vary in capacity.

  - The above new limitations text around access addresses this, and we have added additional text to Methods:

    "The model assumes that all PWID who wish to obtain MOUD treatment are able to do so, and there are no constraints on individual provider treatment capacity."

Reviewer #2

Thank you for the opportunity to review this examination of travel distance scenarios to methadone treatment and the associated influence of syringe sharing risks mediated by an ABM. I really like how the paper makes the benefits of spatial accessibility to methadone more tangible by quantifying reduction in risky behavior and showing how those benefits are located rather tightly around the resources. This is an important *so what* to take away from the research. The use of counterfactual scenarios also really helped to highlight the importance of strategic placement of MOUD resources. I think this work adds value to MOUD accessibility literature, and from a methodological standpoint, the integration of the ABM + accessibility analysis is instructive and creative.

One overarching concern I have for the narrative of this paper is the rather simplistic link that is made between spatial access/low distance and treatment benefits. While I realize the authors are likely trying to showcase possible benefits, I think it is important to address the substantial friction between people (particularly minority population groups) and treatment utilization, so the approaches presented in this paper are situated appropriately within the MOUD problem space.

- We have raised this concern in the limitations section. The data that is used for simulation is predominantly among minoritized community members. We agree that including social phenomena such as degree of medical mistrust, racism and stigma towards treatment are all important to model, but were beyond the scope of this analysis. We now include in the limitation that these unmeasured, potentially impactful forces may further moderate the findings described.

For example, important minority access issues to methadone treatment are mentioned starting on line 89. However, the statement "*though once engaged in treatment*, have similar retention rates to the majority of clients (Elwy et al. 2008)" could mask or misconstrue access issues experienced by minority populations that influence and create substantial inequities of minority populations' induction, adherence, and treatment completion rates when compared to the White population. Guerrero et al. (2022) provides a good overview of this.

Guerrero, Erick G., Hortensia Amaro, Tenie Khachikian, Mona Zahir, and Jeanne C. Marsh. "A Bifurcated Opioid Treatment System and Widening Insidious Disparities." *Addictive Behaviors* 130 (July 1, 2022): 107296. https://doi.org/10.1016/j.addbeh.2022.107296.

- Thank you, this is a great addition to the introduction and provides important context. We have included some of Reviewer 2's comment and citation for Guerrero et al.

Building on this, recent work (Mitchell et al., 2023) suggests that the availability of accessible MOUD providers is not the barrier to MOUD access for urban Black communities—given that consistently across the US at census tract level Black urban populations have substantially greater accessibility to MOUD than White urban communities but Black communities have

worse outcomes as highlighted above by Guerrero. Additionally, the literature has documented how more affluent communities see these treatment resources as negative assets, driving the NIMBY phenomenon to keep these facilities out of their communities.

Mitchell, Penelope, Kevin M. Curtin, and Nicholas R. Magliocca. "Race, Rurality and Geographic Accessibility to Medication for Opioid Use Disorder in the U.S." *Journal of Maps* 19, no. 1 (December 31, 2023): 2270632. https://doi.org/10.1080/17445647.2023.2270632.

While it is not the job of this article to reconcile these complex accessibility and access problems, these issues should at least me noted and mentioned as limitations.

- Thank you, we include these important factors that address race, urbanicity and provider factors in the limitations section, and have included the reference to Michell et al:
    - "Synthetic model population data used in this analysis predominantly represent minoritized community members. The authors acknowledge that including social phenomena such as degree of medical mistrust, racism and stigma towards treatment are all important factors in urban MOUD induction and adherence [55], but are beyond the scope of this analysis. These unmeasured, potentially impactful forces may further moderate the findings described."

Other notes:

- Methods (lines 191-192)—it would be helpful if how distances were calculated (network vs Euclidean) was more clearly stated since the distance thresholds in the analysis are pretty small.
    - Please see above response to Reviewer #1.

- Data discussion (starting at line 424)—I like how this study identifies that targeting the demand population of PWID, as opposed to the general adult population, better offsets syringe exchange. This provides valuable insights in future intervention development—one of the goals of the research. However, much of the PWID data came from syringe service programs which are not available everywhere. Could the authors speak to the challenges of characterizing the PWID population in areas where this type of data is not available to help highlight the value added by this data.
    - Only one of the five datasets is from a large SSP. Furthermore, PWID from five sources shared similar profiles, i.e., SSP participants were not significantly different from NHBS participants. So, data on PWID could be gathered from SSP and non-SSP sources. Also, please note that this approach is generalizable to most urban areas in north America where syringe exchange is available. We have added clarifying text to

METHODS.   Also, please note the description of data sources in the original manuscript:

- "In brief, this includes data from a large syringe service program (SSP) enrollees (n=6,000, 2006-13) [31], the IDU data collection cycles of the National HIV Behavioral Surveillance (NHBS) survey from 2009 (n=545) [32] and 2012 (n=209) [33], and a social network and geography study of young (ages 18-30) PWID (n=164) [34]."
